# Non-neuronal cholinergic stimulation favors bone mass accrual

**DOI:** 10.3389/fphys.2025.1684102

**Published:** 2025-11-03

**Authors:** Faleh Tamimi, Hazem Eimar, Sharifa Alebrahim, Lina Abu-Nada, Garthiga Manickam, Ahmed Ebraheem Al Subaie, Iskandar Tamimi, Monzur Murshed

**Affiliations:** ^1^ Faculty of Dental Medicine and Oral Health Sciences, McGill University, Montreal, QC, Canada; ^2^ School of Dentistry, University of Jordan, Amman, Jordan; ^3^ College of Dental Medicine, Qatar University, Doha, Qatar; ^4^ Department of Oral and Craniofacial Health Sciences, College of Dental Medicine, University of Sharjah, Sharjah, United Arab Emirates; ^5^ Faculty of Medicine, McGill University, Montreal, QC, Canada; ^6^ Genetics Unit, Shriners Hospital for Children, Montreal, QC, Canada

**Keywords:** bone, cholinergic neurons, muscarinic receptors, immune system, IL-17, bone remodeling

## Abstract

**Introduction:**

Non-neuronal cholinergic receptors are expressed in immune cells and their stimulation has been shown to regulate the secretion of several cytokines. Some of these cytokines, such as interleukin-17 (IL-17), IL-23, interferon-γ (IFN-γ) and tumor necrosis factor-α (TNF-α), are known to regulate bone mass. Accordingly, we hypothesize that stimulating cholinergic receptors in non-neuronal cells, such as immune cells, promotes bone mass accrual.

**Methods:**

To test this hypothesis, we used neostigmine, a drug that increases acetylcholine levels by inhibiting acetylcholinesterase activity in peripheral tissues. Female C57BL/6 mice were treated with neostigmine for six weeks, and μCT, histomorphometry, Raman spectroscopy, X-ray diffraction, and mechanical testing were used to analyze bone parameters. A rat model was used to assess bone defect healing and implant osseointegration. Serum cytokines were measured by ELISA, and IL-17 effects on osteoblast proliferation were evaluated *in vitro*.

**Results:**

Here, we show that 6 weeks of neostigmine treatment promotes bone mass accrual in endochondral bones of both the axial and appendicular skeleton in mice. Moreover, the administration of neostigmine for 2 weeks accelerated the healing process of the surgically induced bone defects in rats. The body mass index, body weight, visceral fat pad weight and epinephrine levels in the neostigmine-treated mice were similar to those of saline-treated mice, indicating that neostigmine favored bone mass accrual by acting peripherally rather than centrally. The increased bone mass in the neostigmine-treated mice was caused by an increase in osteoblast proliferation and bone formation rate. We also observed an increase in circulating immunocytokine IL-17 levels in the neostigmine-treated mice. Statistical analysis showed that the increase in serum IL-17 level was associated with the increase in osteoblast number. In agreement with our findings from the *in vivo* experiments, IL-17 treatment increased the proliferation of MC3T3.E1 preosteoblasts *in vitro*, while acetylcholine or neostigmine did not have any significant effect.

**Conclusion:**

Taken together, our findings indicate that peripheral cholinergic stimulation promotes bone mass accrual, in part through IL-17–mediated osteoblast activity. Although the evidence is correlative, these results highlight a potential neuro-immune pathway and suggest new therapeutic directions for enhancing bone formation and regeneration.

## 1 Introduction

Bone remodeling is a lifelong process which is critical to maintain a healthy bone mass. This process involves a balance between bone resorption and bone formation and can be regulated both locally and centrally (Schett and David, 2010). The direct interactions between bone cells (i.e., osteoblasts and osteoclasts) and their interaction with immune cells (i.e., T-cells) regulate the net outcome of bone remodeling locally (Schett and David, 2010; Takaya and nagi, 2007). Centrally, bone remodeling is regulated through the hypothalamic-pituitary-thyroid axis, which coregulates bone, adipose tissue and energy metabolism via the adrenergic sympathetic arm (SNS) of the autonomic nervous system (Kondo et al., 2013; Katayama et al., 2006; Rosen, 2008; Hinoi et al., 2008; Elefteriou et al., 2005; Takeda et al., 2002; Ducy et al., 2000; Eimar et al., 2013). On the other hand, previous studies have provided evidence that the other arm of the autonomic nervous system, the cholinergic parasympathetic nervous system (PNS), also affects bone remodeling (Shi et al., 2010; Bajayo et al., 2012; Tamimi et al., 2012; Tamimi et al., 2018; Eimar et al., 2016).

The main neurotransmitter of the PNS is acetylcholine (Loewi, 1921). In cholinergic neurons, choline acetyl transferase, a cytoplasmic enzyme, synthesizes acetylcholine from acetyl-CoA and choline (Nachmansohn and John, 1945). During neurotransmission, acetylcholine released from the nerve-ends exerts its signalling effects by targeting either nicotinic (α, β, γ, δ and ε) or muscarinic receptors (ChRM 1-5) present in the post-synaptic neurons or non-neuronal cells (Dani and Bertrand, 2007; Caulfield and Birdsall, 1998). The signal is then terminated when acetylcholine is hydrolyzed by acetylcholinesterase, present mainly in the cholinergic nerve synapses and junctions (Taylor and Radic, 1994).

Previous studies have shown that the cholinergic system components (transmitter, enzymes and receptors) are widely expressed in bone. Indeed, cholinergic receptors, both nicotinic and muscarinic, have been identified on the membranes of human primary bone cells, mesenchymal stem cells, osteocytes, osteoblasts and osteoclasts (Shi et al., 2010; Bajayo et al., 2012; Liu et al., 2011; En-Nosse et al., 2009; Walker et al., 2001; Paic et al., 2009). However, the first proof of cholinergic regulation of bone mass came from an animal study in which it was shown that mice lacking muscarinic receptor 3 (ChRM3) globally present a low bone mass phenotype caused by decreased bone formation and increased bone resorption (Shi et al., 2010). These mice showed an increased sympathetic tone, suggesting a neuronal role for ChRM3 in the regulation of bone mass (Shi et al., 2010). Later Bajayo et al. (2012) showed that nicotinic (cholinergic) nerve fibers innervate bone tissue and that their activity regulates bone mass through signaling mediated by central interleukin- (IL-1), a proinflammatory cytokine produced by brain neurons (Bajayo et al., 2012). Indeed, mice with blocked central IL-1 signaling exhibit markedly reduced skeletal acetylcholine levels, and these mice are osteoporotic due to the decrease of apoptosis in osteoclasts (Bajayo et al., 2012). In addition, we showed that stimulating the activity of the cholinergic system centrally (neuronal tissues level) by the administration of a central-acting cholinergic agonist promotes bone mass accrual in wild-type mice (Eimar et al., 2015). However, the role of peripheral (non-neuronal) activity of the cholinergic system on bone mass was not clarified in these studies.

The cholinergic pathway is also known to regulate the immune system through a newly discovered cholinergic-immune pathway (Pavlov et al., 2003). The cholinergic-immune pathway plays a critical role in controlling systemic and local inflammatory processes via the peripheral cholinergic receptors in immune cells (Pavlov et al., 2003; Elefteriou, 2018). Cholinergic receptors are expressed in the immune cells, such as T-lymphocytes and macrophages, and their activation has been shown to regulate the secretion of several cytokines (Tracey, 2002; Kawashima and Fujii, 2003a; Tayebati et al., 2002; Fujii et al., 1999; Kawashima and Fujii, 2003b). Some of these cytokines, such as IFN-γ and TNF-α, are known to regulate bone mass accrual by affecting osteoclast activity; whereas one cytokine in particular, IL-17, has the ability to stimulate osteoblast proliferation (Schett and David, 2010; Tracey, 2002; Kawashima and Fujii, 2003a; Tayebati et al., 2002; Fujii et al., 1999; Kawashima and Fujii, 2003b; Lee, 2013).

Based on the above findings, we hypothesize that cholinergic activity regulates bone metabolism through the immune system. One way of stimulating the cholinergic system peripherally (non-neuronal tissues) is by the administration of specific cholinergic agonists such as acetylcholinesterase inhibitors (AChEIs), which are unable to cross the blood-brain barrier. AChEIs stimulate that cholinergic activity by increasing the levels of acetylcholine in the synaptic space. One of these FDA-approved peripherally acting AChEIs is neostigmine (Janowksy et al., 1972; Yamamoto et al., 1995; Pepeu and Giovannini, 2009), which has also been shown to modulate the activity of the immune system (Pohanka et al., 2012; Fleming et al., 1991). Accordingly, our specific aim is to investigate the effects of neostigmine administration on bone mass, and whether these potential effects are driven by the activity of the immune system.

In this study, we provided evidence for the previously unexplored role of nonneuronal (peripheral) cholinergic stimulation on bone accrual. First, we showed that a cholinergic agonist (neostigmine) acting peripherally improves bone qualities in female mice by increasing the bone formation rate and the number of osteoblasts. Interestingly, the increase in bone mass following neostigmine treatment was associated with the serum level of immunocytokine IL-17. In agreement with our findings from the *in vivo* experiments, IL-17 treatment increased the proliferation of MC3T3.E1 preosteoblasts *in vitro*, while acetylcholine or neostigmine had no significant effect. These results indicate that the peripheral cholinergic stimulation of non-neuronal tissues by the administration of neostigmine promotes bone mass accrual indirectly through the immune system.

## 2 Materials and methods

All experimental procedures of this study were performed following an animal use protocol approved by the Animal Care Committee of McGill University.

### 2.1 Materials

For mice experiments, we used 5-week-old female wild-type C57BL/6J mice (n = 24) procured from Jackson Laboratory (Bar Harbor, MA). Used medications for intraperitoneal injections were neostigmine and normal saline obtained from Sigma-Aldrich (St. Louis, MO, United States). Following animal euthanasia, the bone phenotype of the mice was analyzed using three-dimensional micro computed tomography (SkyScan 1072 machine, Bruker-Microct, Kontich, Belgium), Dual X-ray Absorptiometry (PIXIMUS bone densitometer, GE Medical Systems, Schenectady, NY, United States), Raman spectrophotometer (Senterra, Bruker, Karlsruhe, Germany), and X-ray diffraction (D8-Discover/GADDS, Bruker, Karlsruhe, Germany). Materials used for bone histomorphometry analyses were calcein solution (0.25% calcein and 2% NaHCO_3_ dissolved in 0.15 M NaCl), 4% PFA/PBS, methyl methacrylate resin, and the following stains: von Kossa/van Gieson, toluidine blue, and tartrate-resistant acid phosphatase (TRAP). Mechanical testing included the following machines: Instron 5569 (Instron Corp., Canton, MA, United States) and Vickers microhardness indenter machine (Clark CM100 AT, HT-CM-95605, Shawnee Mission, KS). Analyses of metabolic parameters included serum leptin (ELISA kit, Life Technologies, Gaithersburg, MD) and insulin (ELISA kit, B-Bridge International, BioCat, Cupertino, CA), C-terminal telopeptides of type I collagen (ELISA kit, RatLaps EIA (IDS)), IL-17, TNF-α, and IFN-γ (ELISA kits, eBioscience, San Diego, CA), urinary catecholamine (ELISA kit, BlueGene, Biotech, Shanghai, China), and creatinine (ELISA kit, Quidel Corporation, San Diego, CA). Materials for Locomotor activity were an open field transparent box (30 × 30 × 60 cm) and a camera.

For rat experiments, we used 10 to 12-week-old Sprague-Dawley rats (n = 18) procured from Charles River Laboratories (Montreal, QC, Canada). The following materials used for surgical procedures: surgical instruments [handpiece drill (Stryker, Hamilton, ON), cylindrical burr (1.5 mm in diameter)), cylindrical burr (2.5 mm in diameter) and Ti implants, 5-0 monocryl sutures], isoflurane anesthetic agent, chlorhexidine scrub, Carprofen (Pfizer Animal Health, Montréal, QC). Used medications for intraperitoneal injections were neostigmine and normal saline obtained from Sigma-Aldrich (St. Louis, MO, United States). Reserving agents used were paraformaldehyde solution 4% in PBS (Santa Cruz Biotech, Dallas, TX) and 10% neutral buffered Formalin (Richard Allan Scientific, Kalamazoo, MI). Bone accrual analyses were performed using three-dimensional μCT (SkyScan 1072 machine, Bruker-Microct, Kontich, Belgium). Materials used for histomorphometry analysis were polymethyl methacrylate histological resin (Technovit 9100, Heraeus Kulzer, Wehrheim, Germany), a diamond saw (SP1600, Leica Microsystems GmbH, Wetzlar, Germany), basic fuchsine and methylene blue stains, and an optical microscope (Carl Zeiss Microscopy, GmbH, Germany).

For the *in vitro* experiments, we used MC3T3.E1 preosteoblasts cell line (ATCC, Manassas, VA, United States). Medications used were acetylcholine, muscarine, nicotine, neostigmine, IL-17, and saline obtained from Sigma-Aldrich (St. Louis, MO, United States). Five-Bromo-2′-Deoxyuridine (BrdU) labeling kits (Abcam, Toronto, ON) were used to assess the proliferation rates of the cell lines.

### 2.2 Animals

Five-week-old female wild-type C57BL/6J mice were used in this study. Female mice were chosen because they are more prone to metabolic and bone diseases (i.e., osteoporosis) than males (Guggenbuhl, 2009). All mice were kept in a pathogen-free standard animal facility, maintained under a 12-h light-dark cycle with ad libitum access to food and water. Animals were randomly assigned to two groups (SAL = saline control, NEO = neostigmine-treated), with n = 12 animals per group. Group 1 was injected intraperitoneally (IP) with neostigmine (NEO) (0.08 mg/kg/day), and group 2 was injected IP with normal saline (SAL) (0.2 mL/day) as a control group. The mice were euthanized 6 weeks after the start of drug injection. Neostigmine was administered intraperitoneally at a dose of 0.08 mg/kg once daily. This dose was selected based on previously reported effective and safe ranges (0.05–1 mg/kg i.p.) in rodent models, which produced consistent systemic pharmacological effects without toxicity (Yoon et al., 2005; Pohanka et al., 2012; Luo et al., 2018).

Eighteen female, 10 to 12-week-old Sprague-Dawley rats weighing between 200 and 250 g were used. All rats were kept in a pathogen-free standard animal facility, maintained under a 12-h light-dark cycle with ad libitum access to food and water. Animals were allowed to acclimatize to this environment for at least 1 week prior to surgical intervention.

All rats were submitted to surgical procedures as previously described (Al Subaie et al., 2015). During the surgical intervention, the surgeon was blinded to group allocation, and all surgical interventions were performed by one surgeon using standardized instruments (i.e., burr and Ti implants) to ensure consistent bone defects and implant placement among all animals. Briefly, the animals were anesthetized with isoflurane (3%-5% at induction, 2%-2.5% at maintenance period). After the animal showed signs of being fully anesthetized, the legs were shaved and disinfected with chlorhexidine scrub. The muscle was dissected, and the periosteum was conserved. A unicortical defect was produced using a cylindrical bur (1.5 mm in diameter) adapted to a handpiece drill under constant saline irrigation. A custom-made (1.5 mm × 2.0 mm in depth) titanium implant was placed in the left defect. The incision was closed using 5-0 monocryl sutures. The same procedure was done on the contralateral tibia, but the defect was 2.5 mm in diameter and was left empty. Carprofen was injected (5-10 mg/kg) subcutaneously (SC) into the rats 30 min prior to surgery and 24 h after surgery for 2 days in order to provide analgesia to the rats. After surgical intervention, the rats were randomly divided into 2 assigned groups of 9 each and treated daily with one specific drug for 6 weeks. Group 1 was injected intraperitoneally (i.p.) with neostigmine (0.08 mg/kg), and group 2 was injected i.p. with normal saline (0.2 mL) as a control group. The rats were assessed daily for signs of toxicity and infections. The rats were euthanized 2 weeks after the start of drug injection. The 2-week duration of treatments was planned in order to be able to see the differences, if any, between the 2 rat groups in bone defect healing and implant osseointegration before the total healing/osseointegration occurs. Euthanasia method was approved by the McGill University Facility Animal Care Committee and according to McGill SOP #301. Rats were anesthetized with 5% isoflurane, then euthanized with gradual-fill CO_2_ (30%–40% chamber volume/min) until breathing ceased. Animals remained in CO_2_ for ≥2 min, and death was confirmed by bilateral thoracotomy. Following animal euthanasia, right and left tibiae were retrieved from each rat. Right tibias were preserved in Paraformaldehyde solution 4% in PBS, while the left tibias were preserved in 10% neutral buffered Formalin.

### 2.3 Locomotor activity

Locomotor activity of the different treated mice groups was evaluated using an open field box. This test is commonly used to assess the effects of medications on animal movement, mood, and anxiety, which are controlled by the central nervous system (Gould et al., 2009). The open field box consisted of a transparent plastic box (30 × 30 × 60 cm). The floor of the box was divided into nine squares. Mice were placed individually in the box for 5 min, and their spontaneous behaviors were video recorded by a camera located above the plastic box. The number of squares crossed by each mouse during this period was counted.

### 2.4 Analyses of metabolic parameters

Body weight and abdominal fat pad weight were assessed for each mouse. Blood and urine samples from each mouse were collected before *euthanasia*. Serum leptin and insulin levels were measured using commercially available ELISA kits. Urinary catecholamine contents were measured in acidified spot urine samples, and creatinine was used to standardize between urine samples. Serum C-terminal telopeptides of type I collagen (CTX) level and serum IL-17, TNF-α, and IFN-γ levels were assessed using commercially available ELISA kits.

### 2.5 3-dimensional -micro computed tomography (μCT) analysis

For mice experiments, μCT analyses were performed as previously described (Duque et al., 2011). Briefly, the collected proximal left tibiae and axial (represented by lumber vertebra number 5 (L5)) bones of each mouse were scanned using a SkyScan 1072 machine adjusted to the following parameters: 50 kV x-ray energy, 200 μA x-ray current, 0.5 mm Aluminum filter, 5 μm image pixel size, and 1000 pixel field width resolution. The analysis of the tibia included a region of interest of 2.3 mm distal to the growth plates, whereas the analysis of the vertebra included the whole body and was analyzed using bone-analysis software (Version 2.2f, Skyscan, Kontich, Belgium). The following three-dimensional morphological parameters were evaluated: bone volume fraction (BV/TV), trabecular thickness (Tb.Th), trabecular separation (Tb.Sp), trabecular number (Tb.N), and cortical thickness (Ct.Th) (Bouxsein et al., 2010).

For rat experiments, μCT analysis was conducted to assess bone healing in the defects as previously described (Al Subaie et al., 2015). Collected right tibias with empty defects were scanned using a μCT scanner set at the same parameters described above in the mice study. The area of the bone defect was determined and included in the region of interest (ROI). The volume of the defect was determined by subtracting the bone volume from the tissue volume.

### 2.6 Dual X-ray absorptiometry (DXA)

For mice experiments, bone mineral density (BMD) of the left tibia collected from different treatment groups was measured using a PIXIMUS bone densitometer. The DXA parameters were adjusted according to previous work in the field (Duque et al., 2011).

### 2.7 Raman analysis

For mice experiments, crystallinity index analysis on the mid-shaft of the left tibia was conducted by means of Raman spectroscopy. A Raman spectrophotometer equipped with a 785 nm diode laser (of 50 mW power) coupled with an optical microscope (Olympus Optical Co., Hamburg, Germany). The Raman spectrophotometer parameters were adjusted according to previous work in the field (Eima et al., 2012). Crystallinity index was calculated based on the band width at the half peak intensity of the ν_1_PO_4_^−3^ band at 960 cm^−1^.

### 2.8 X-ray diffraction (XRD)

For mice experiments, the left tibia of each mouse was manually crushed into powder, defatted with Acetone (Sigma–Aldrich, Oakville, Ontario), and left to dry at ambient temperature for 48 h. The bone powder specimens were submitted to XRD. The XRD parameters were adjusted according to previous work in the field (Eimar et al., 2012). DIFFRACplus EVA software (Bruker AXS, Karlsruhe, Germany) was used to analyze the XRD spectra. Crystal dimensions along a and c-axes were calculated from the peak broadening of the powder X-ray diffraction peaks (002) and (310), respectively, according to the Scherrer formula (Eimar et al., 2012).

### 2.9 Mechanical testing

For mice experiments, a three-point breaking test was performed on the midshaft of the mice right tibiae using Instron 5569 machine (Duque et al., 2011). The span of two support points was 10 mm, and the deformation rate was 1.0 mm/min. The extrinsic parameters, stiffness and ultimate force, were calculated from the resulting load-displacement curves.

A Vickers microhardness indenter machine was employed on the cortical bone of the resin-embedded lumber vertebral bodies (Mousny et al., 2008). The indentation load was adjusted to 10 g per 10s. A computer software (Vision PE 3.5, Clemex Technologies Inc., Shawnee Mission, KS) was used to measure the microhardness value at the site of indentation from images captured with a built-in camera. Due to the variations in microhardness within the cortical bone, 10 readings were performed for each cortical bone sample with a minimum distance apart of 50 μm (Eimar et al., 2012). The microhardness profile of each cortical bone was obtained by calculating the average of the 10 readings.

### 2.10 Bone histomorphometry

For mice experiments, histomorphometric analyses were performed as previously described (Marulanda et al., 2013; Khavandgar et al., 2011). Briefly, calcein solution (0.25% calcein and 2% NaHCO_3_ dissolved in 0.15 M NaCl) was injected twice IP in mice (10 μL/g body weight) at an 8-day interval. Mice were euthanized 2 days after the second calcein injection. Lumbar vertebrae were fixed for 24 h in 4% PFA/PBS, dehydrated in graded ethanol series, embedded in methyl methacrylate resin according to standard protocols, and sectioned (7-μm thickness). The undecalcified sections of the lumber vertebra were stained by von Kossa/van Gieson, toluidine blue, and tartrate-resistant acid phosphatase (TRAP). Stained bone sections were analyzed for bone volume-to-tissue volume ratio (BV/TV), osteoblast count, osteoclast count and bone formation rate-to-bone surface (BFR/BS) using the Osteomeasure software (Osteometrics, Inc.). All histological images were captured using a camera (DP72; Olympus), acquired with DP2-BSW software (XV3.0; Olympus), and processed using Photoshop (Adobe).

For rat experiments, osseointegration analysis was conducted as previously described (Al Subaie et al., 2015). All left tibias (with implants) were dehydrated before embedding them in polymethyl methacrylate histological resin, sectioned into histological slides (20 µm thickness) using a diamond saw and stained using basic fuchsine and methylene blue. Digital optical micrographs of the histological sections were recorded with an optical microscope. Histomorphometric measurements were performed using ImageJ software (Wayne Rasband; National Institute of Health, Bethesda, MD). Implant osseointegration was defined as bone implant contact area (BIC) and was calculated by dividing the bone-covered implant perimeter (BIP) by the total implant perimeter (TIP) as shown in this equation: BIC = BIP/TIP%.

### 2.11 BrdU *assay*

MC3T3.E1 preosteoblast cell line was used for the cell culture studies. Preosteoblast cultures were treated either with acetylcholine (100 μM), muscarine (10 μM), nicotine (10 μM), neostigmine (0.08 mg/mL), IL-17, or saline (control). Proliferation rate in cell culture studies was assessed with 5-Bromo-2′-Deoxyuridine (BrdU) labeling using a commercially available kit (Abcam, Toronto, ON).

### 2.12 Statistical analyses

All results from the animal studies are presented as descriptive outcomes (mean ± SD). The number of animals per group was n = 12. The normality of the data was verified using the Shapiro–Wilk test. For pairwise comparisons, statistical analyses were performed using Student’s two-tailed unpaired t-test. For analyses involving more than two groups, one-way analysis of variance (ANOVA) was used, followed by Tukey’s post hoc test to correct for multiple comparisons. The associations of IL-17 with BV/TV and the number of osteoblasts were assessed using Pearson correlation analysis. For the *in vitro* studies, results are presented as the mean ± SD of triplicate experiments (three separate experiments) ± SD. All calculations were performed using Origin7 software package (OriginLab, Northampton, MA, United States) and Statistical Package for the Social Sciences (SPSS) 23.0v for Windows (SPSS Inc., Chicago, IL, United States). In all experiments, a value of *p < 0.05* was considered significant as indicated by a single asterisk.

## 3 Results

### 3.1 Effects of neostigmine on bone microstructure and mechanical properties in mice

We compared the bone phenotypes of 5-week-old female C57BL/6 mice treated for 6 weeks with either neostigmine or saline (control). Axial (vertebra) and long (tibia) bones were analyzed using μCT, histomorphometry, Raman spectroscopy, X-ray diffraction (XRD), and mechanical testing.

**FIGURE 1:**
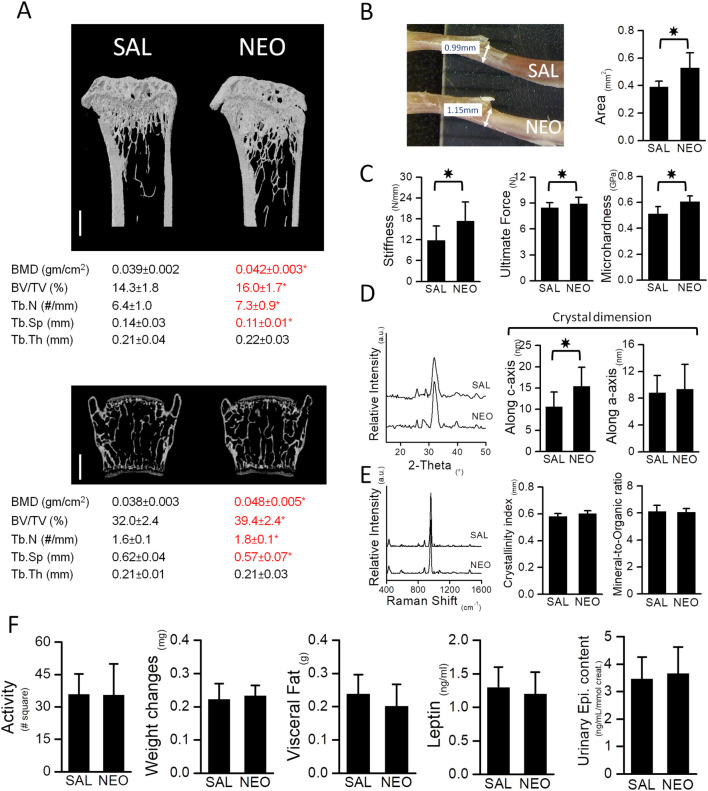
Neostigmine, a peripherally acting AChEI, favors bone mass and enhances the biomechanical properties of bones. **(A)** μCT images of tibia (top) and vertebra (bottom). Scale bars, 500 μm. Neostigmine-treated mice had higher bone mineral density (BMD), bone volume (BV/TV), trabecular number (Tb.N) and lower spacing between trabeculae (Tb.Sp) compared to the saline-treated mice. No significant change in trabecular thickness (Tb.Th) was observed between the two groups. **(B)** A photograph showing the diameter of tibiae obtained from mice treated with saline (SAL) (top) or neostigmine (NEO) (bottom). Cross sectional area of mid shaft tibiae in neostigmine-treated mice were larger compared to the saline-treated mice. **(C)** Three-point-bending and Vickers’ microhardness tests showed higher bone stiffness, ultimate force and microhardness in neostigmine-treated mice compared to control. **(D,E)** XRD and Raman spectroscopy analyses revealed that bones of the neostigmine-treated mice had larger crystal dimensions along the c-axis with no changes in crystallinity index and mineral-to-organic ratio compared to the control group. **(F)** No significant differences were observed in locomotor activity, body weight, body visceral fat and serum leptin levels or urinary epinephrine levels between the neostigmine- and saline-treated mice, indicating that neostigmine did not alter the SNS tone. (n = 9/group). Data are mean ± SD. *p < 0.05.

Mice treated with neostigmine had higher bone volume/tissue volume (BV/TV), trabecular number (Tb.N), and bone mineral density (BMD) compared to saline-treated mice (Figure 1A). With the increase in trabecular number, trabecular spacing (Tb.Sp) decreased significantly in the experimental group (Figure 1A). Analyses of bone mechanical and physical properties showed higher stiffness, ultimate force, microhardness, and increased crystal dimensions in the neostigmine-treated bones compared to controls (Figures 1B–D). A slight, non-significant trend toward an increased crystallinity index was observed in the NEO group compared with controls (*p > 0.05*), while the mineral-to-organic ratio remained unaltered between the two groups. (Figure 1E).

### 3.2 The effect of neostigmine on metabolic parameters

No significant differences were observed in locomotor activity, body weight, body fat, serum leptin and insulin levels, or urinary epinephrine levels between the neostigmine- and saline-treated mice (Figure 1F).

### 3.3 The effects of neostigmine on bone healing and implant osseointegration in rats

**FIGURE 2:**
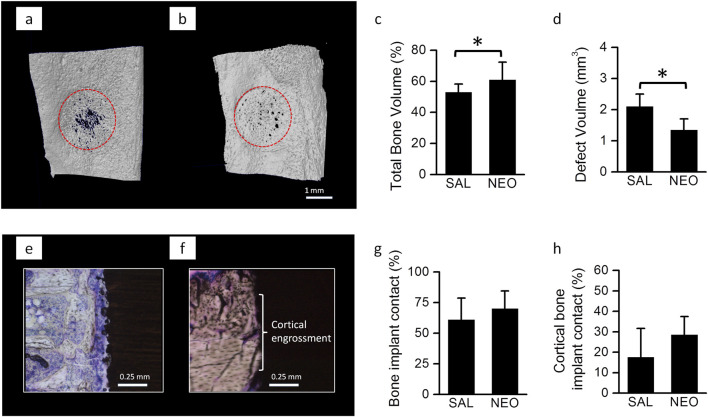
Bone healing and implant osseointegration in neostigmine-treated rats: **(a)** 3-D reconstructions of 2-week old bone defects on medial surface of the tibiae in rats treated with neostigmine (NEO) and saline (SAL) showing that neostigmine improved healing of the bone defects. **(b,c)** µ-CT analyses of the bone defects showing that neostigmine-treated rats had higher bone volume and thus, smaller defect volume compared to saline-treated rats (n = 9/group). **(d)** Longitudinal histological sections of Ti implant placed in rats tibiae showing the bone-implant contact in neostigmine- and saline-treated rats. **(e–h)** Histomorphometric analyses for bone-implant contact showed no significant difference between neostigmine- and saline-treated rats. Data are mean ± SD. *p < 0.05.

A parallel *in vivo* study in rats was conducted to assess whether 2 weeks of neostigmine treatment favors bone healing and osseointegration. Neostigmine-treated rats showed significantly accelerated healing of the bone defect (higher bone volume and smaller defect size) compared to saline-treated rats (Figures 2a–c). No significant differences were observed in titanium implant osseointegration between the two groups (Figures 2d–f).

### 3.4 The effect of neostigmine on bone histomorphometry and bone turnover markers

**FIGURE 3:**
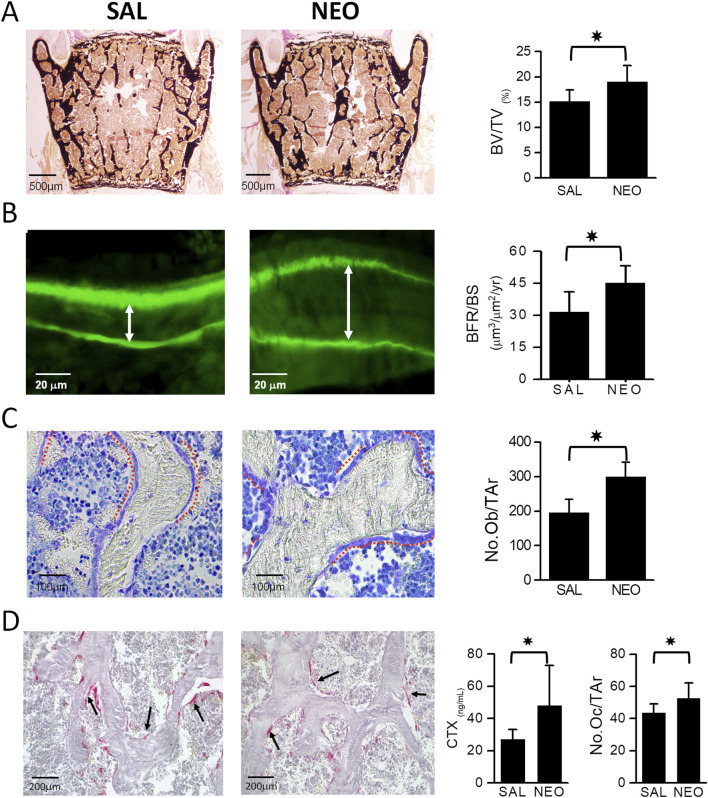
Neostigmine treatment increases bone mass by stimulating bone formation rate. **(A)** Von Kossa and van Gieson-stained lumbar vertebra sections showing that mice treated with neostigmine (NEO) had higher BV/TV compared to saline (SAL). **(B)** Calcein double labeling shows a significantly increased bone formation rate over bone surface (BFR/BS) in neostigmine-treated mice compared to controls. The white arrows show the distance between calcein double labels. **(C,D)** Toluidine blue- and tartrate-resistant acid phosphatase (TRAP)- staining of lumbar vertebra sections demonstrates that the osteoblast and osteoclast numbers were significantly higher in neostigmine-treated mice than in control mice. In agreement with the increase in osteoclast number, the serum collagen C-terminal telopeptide (CTX) was increased in the neostigmine-treated group compared to the saline-treated group. Data are mean ± SD. (n = 9). *p < 0.05.

Histomorphometric analyses of lumbar vertebrae in mice confirmed the increase in BV/TV in neostigmine-treated animals and showed that it was accompanied by an increase in bone formation rate over bone surface (BFR/BS) (Figures 3A,B). Both osteoblast and osteoclast numbers were significantly higher in neostigmine-treated mice than in controls, consistent with an increase in bone turnover. Serum collagen C-terminal telopeptide (CTX) levels were also increased (Figures 3C,D).

### 3.5 The effect of neostigmine on osteoblast proliferation and immune-related cytokine responses

**FIGURE 4:**
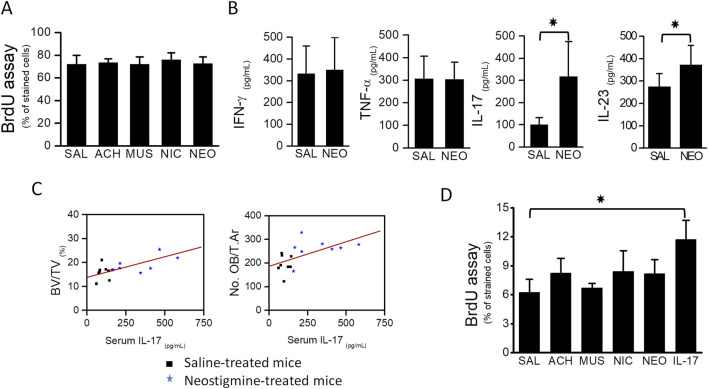
Osteoblast proliferation was stimulated by the immunocytokine IL-17. **(A)** BrdU assay showing that proliferation of MC3T3.E1 preosteoblasts was not stimulated upon treatment with cholinergic ligands, acetylcholine, muscarine, nicotine and an AChEI, neostigmine. **(B)** Graphs showing serum levels of TNF-α, IFN-γ, IL-17 and IL-23 in neostigmine- and saline-treated mice. Serum IL-17 level was 3-fold higher in the neostigmine-treated group compared to the saline-treated group. Serum level of IL-23 was also increased in neostigmine-treated mice. **(C)** Graphs showing the associations between serum IL-17 level, obtained from neostigmine- and saline-treated mice, and bone volume (R = 0.624; p = 0.009) and osteoblasts number (R = 0.579; p = 0.029). **(D)** Proliferation rates of different MC3T3.E1 preosteoblast cultures treated for 4 days with either acetylcholine, neostigmine, IL-17 or saline (control) were assessed by BrdU analysis assay. As illustrated, the proliferation of MC3T3.E1 preosteoblasts treated with IL-17 was significantly higher than those treated with saline (control). Data are mean ± SD. *p < 0.05.

To examine potential mechanisms, we performed *in vitro* proliferation assays using MC3T3.E1 preosteoblasts. Four-day treatments with neostigmine, acetylcholine, nicotine (nicotinic receptor agonist), or muscarine (muscarinic receptor agonist) did not affect proliferation as measured by BrdU incorporation (Figure 4A).

Considering the known influence of cholinergic stimulation on immune cell activity, we measured serum TNF-α, IFN-γ, IL-17, and IL-23 levels in neostigmine-treated mice. IL-17 increased threefold and IL-23 levels increased slightly relative to controls, while TNF-α and IFN-γ were unchanged (Figure 4B). Correlation analysis revealed a significant positive association between serum IL-17 levels and both BV/TV and osteoblast numbers (Figure 4C), whereas IL-23 showed no significant associations with these parameters. *In vitro*, IL-17 treatment for 4 days significantly increased MC3T3.E1 preosteoblast proliferation, while acetylcholine or neostigmine did not (Figure 4D).

## 4 Discussion

Our data demonstrate that peripheral cholinergic stimulation with neostigmine promotes bone mass accrual and improves bone quality, without altering sympathetic tone or systemic metabolic parameters. The observed increases in BV/TV, trabecular number, bone strength, microhardness, and crystal dimensions indicate a genuine anabolic effect on the skeleton.

Our data showing a stimulatory effect of neostigmine on the bone mass is in agreement with that of Bajayo et al, reporting an increase of bone mass in pyridostigmine-treated mice (Bajayo et al., 2012). However, treatment of mice with pyridostigmine, a reversible AChEI, has been shown to result in the apoptosis of osteoclasts, without affecting bone formation by osteoblasts (Bajayo et al., 2012). On the contrary, our data show a bone anabolic effect of neostigmine as a result of increased bone formation by osteoblasts. The discrepancy between the effects of these two different classes of AChEIs on the dynamic parameters of bone remodeling could be explained by their differential mode of action in the target tissues. This explanation is supported by the observation that, pyridostigmine has fewer muscarinic effects compared to neostigmine (Tormoeh et al., 2008; Biller, 2009; Keys and Blume, 1991).

Our *in vivo* experiments in mice showing unaltered sympathetic activity upon neostigmine treatment indicated that the observed increase of osteoblast number in these mice was solely regulated by a peripheral non-neuronal mechanism. Cholinergic receptors are expressed by osteoblasts, and thus their stimulation may boost osteoblast proliferation and bone formation. In order to investigate the direct effect of cholinergic stimulation on osteoblast proliferation, we performed cell culture experiments using MC3T3.E1 preosteoblasts. As shown in our results, 4-day treatment of these cells with neostigmine, acetylcholine, nicotine (nicotinic receptor agonist), or muscarine (muscarinic receptor agonist) did not affect their proliferation as examined by BrdU assay. These results rule out the possibility of a direct effect of cholinergic stimulation on osteoblast proliferation and explain the observed increase in osteoblast number following neostigmine treatments *in vivo.*

Considering the known effects of cholinergic stimulation on the paracrine activities of immune cells (Tracey, 2002; Kawashima and Fujii, 2003a; Tayebati et al., 2002; Fujii et al., 1999; Kawashima and Fujii, 2003b), we measured the serum levels of sevexplain the increase in osteoblast numbereral pro-osteogenic cytokines, e.g., TNF-α, IFN-γ, IL-17 and IL-23 in neostigmine-treated mice. Neostigmine-treated mice showed a 3-fold increase in IL-17 and only a modest increase in IL-23 levels in comparison to the control. No significant differences in the levels of TNF-α or INF-γ were observed. Accordingly, our data suggests that there is a stimulation of immune cells upon neostigmine treatment.

The increase in IL-23 has been previously shown to stimulate osteoclast proliferation (Adamopoulos et al., 2011; Chen et al., 2008), which might explain the observed increase in osteoclast number in neostigmine-treated mice. However, changes in IL-23 could not explain the observed increase in bone volume and osteoblast numbers following neostigmine treatment, as the associations between serum levels of IL-23 with either BV/TV (correlation coefficient [R] = 0.435; p-value = 0.157) or osteoblast number (R = 0.435; p-value = 0.157) were not significant. However, future *in vitro* and *in vivo* studies will have to be performed to investigate the role of IL-23 in bone metabolism.

IL-17, which is primarily produced by the cells of the macrophage/monocyte lineage, more specifically by the T-helper 17 cells, has been shown to affect both osteoclast and osteoblast numbers. For instance, IL-17 is able to stimulate osteoclastogenesis by upregulating the expression of RANKL via the osteoblasts (Kotake et al., 1999; Moon et al., 2012). On the other hand, recent evidences have linked the IL-17 with osteoblasts proliferation (Lee, 2013; Huang et al., 2006; Ahmed and Gaffen, 2010; Goswami et al., 2009; Huang et al., 2009). It has been shown that IL-17 can stimulate the differentiation of mesenchymal stem cells towards osteoblasts and away from adipocytes (Lee, 2013; Huang et al., 2006; Ahmed and Gaffen, 2010; Goswami et al., 2009; Huang et al., 2009). Moreover, IL-17 has been found to enhance the differentiation of MC3T3-E1 pre-osteoblasts (Huang et al., 2006) and stimulate the expression of mature bone markers (Collagen-1, Collagen-2, bone sialoprotein and osteocalcin) in cell culture studies (Nam et al., 2012). Accordingly, the significant increase in the serum level of IL-17 might explain the increase in osteoblast number in neostigmine-treated mice.

The strong positive correlation between serum IL-17 levels and bone parameters in neostigmine-treated mice, together with the ability of IL-17 to stimulate osteoblast proliferation in vitro, suggests that cholinergic stimulation promotes bone formation indirectly through IL-17–mediated immune signaling. Rather than acting directly on osteoblasts, acetylcholine likely modulates immune cell activity to increase IL-17 production, which in turn enhances osteoblast proliferation and bone accrual. These findings identify IL-17 as a critical intermediary linking local cholinergic activity to bone anabolic responses and underscore the complex neuroimmune regulation of bone remodeling. Nonetheless, these findings remain associative, and definitive proof will require further mechanistic studies, such as immune cell depletion or targeted ablation of IL-17–producing subsets like Th17 cells.

**FIGURE 5:**
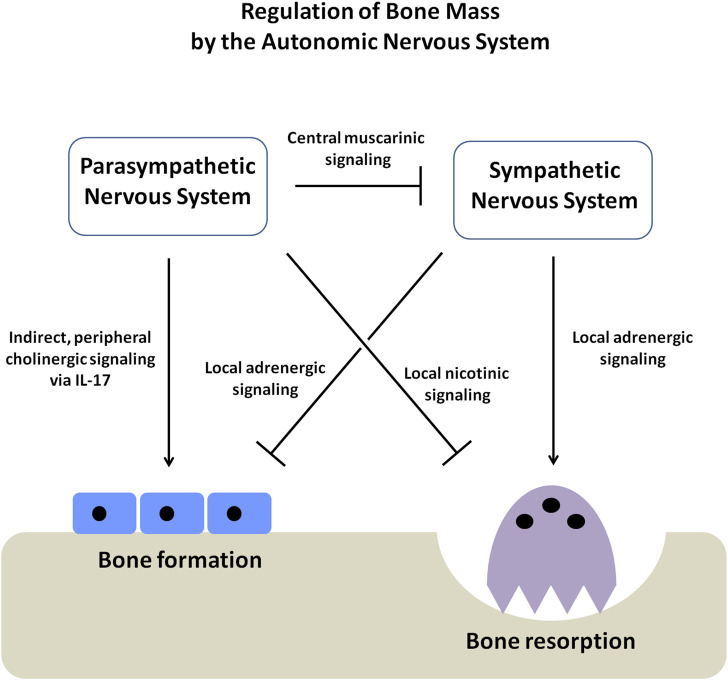
Schematic diagram showing the regulation of bone mass by the autonomic nervous system (ANS). The autonomic nervous system has two arms, the sympathetic (SNS) and parasympathetic nervous systems (PNS), which function in an opposing yet complementary manner. The SNS inhibits osteoblast proliferation and bone formation. At the same time, it promotes osteoclast-mediated bone resorption, causing a net loss of bone mass. On the other hand, the PNS favors bone mass accrual through three different axes; *firstly*, by suppressing the SNS tone through the activity of muscarinic receptors within the CNS, *secondly,* by directly stimulating the apoptosis of osteoclasts through the activity of the nicotinic receptors and *thirdly,* by stimulating osteoblast proliferation and bone formation by elevating the secretion of immunocytokine IL-17.

The findings of this study add important components to the existing model of the regulation of bone mass by the autonomic nervous system (Figure 5) (Eimar et al., 2013). The SNS favors bone mass loss by inhibiting the osteoblast-mediated bone formation and increasing the osteoclast-mediated bone resorption through the local adrenergic activity (Kondo et al., 2013; Katayama et al., 2006; Rosen, 2008; Hinoi et al., 2008; Elefteriou et al., 2005; Takeda et al., 2002; Ducy et al., 2000; Eimar et al., 2013). The PNS appears to favor bone mass accrual through both central and local pathways. The central pathway affects bone mass by suppressing the sympathetic tone through the activity of muscarinic receptors (e.g., ChRM3) expressed in the locus coeruleus (Shi et al., 2010). On the other hand, it has been shown that the local pathway prevents bone resorption by signaling through the nicotinic receptors present in the osteoclasts (Bajayo et al., 2012). Our current study provides further evidence of a local effect of cholinergic signaling on bone mass accrual by showing that cholinergic signaling modulates the peripheral non-neuronal secretion of immunocytokines, promoting osteoblast proliferation and bone formation indirectly.

Although it is well known that the parasympathetic nervous system regulates the activity of the immune cells (Czura and Tracey, 2005). Until recently, the influence of the nervous system or the immune system on bone mass regulation has been assessed as two independent mechanisms (Schett and David, 2010). Our work demonstrates that bone mass is regulated through a neuro-immune pathway, in which the nervous system modulates bone remodeling indirectly by acting on the immune cells. Further work is needed to identify the mediators regulating the crosstalk among the nervous, immune, and skeletal systems and to elucidate the possible mechanisms underlying this novel axis of bone regulation.

Our research revealed an increase in serum IL-17 following neostigmine administration, but we did not conduct a phenotypic evaluation of the immune cells that synthesize it. Although direct measurements of Th17 and γδ-T cells were not performed, these cells were established as the predominant producers of IL-17, and cholinergic signals controlled their activities (Nechanitzky et al., 2023; Roark et al., 2008). Additional studies combining flow cytometry and immunohistochemistry with molecular biology approaches are needed to determine whether the elevated IL-17 levels in neostigmine-treated mice originate from Th17 cells or γδ T cells.

Furthermore, although our *in vitro* experiments revealed that IL-17 directly induces pre-osteoblast proliferation, these studies used monocultures that do not account for interactions between immune cells and osteoblasts. Future studies using co-culture systems that incorporate immune cells will be crucial for verifying that cholinergic stimulation induces IL-17 release from immune cells and for defining the paracrine mechanisms linking the immune and skeletal systems.

It is also worth noting that this study was conducted using young female mice, which exhibit increased sensitivity of their bones to bone turnover and bone loss, thereby allowing for a more pronounced observation of the cholinergic effects on bone physiology. However, excluding male and older subjects will limit the generalizability of the findings. Further studies with both sexes and all age groups will be needed to validate these findings.

## 5 Conclusion

Our findings expand the current model of autonomic regulation of bone mass. The sympathetic nervous system promotes bone loss through adrenergic signaling, while the parasympathetic system supports bone accrual via central and local pathways. Our study provides additional evidence for a neuro-immune axis, in which local cholinergic signaling stimulates immune cells to secrete IL-17, which in turn promotes osteoblast proliferation and bone formation. This integrated pathway highlights novel targets for bone anabolic therapies. Although our findings are correlative, they are consistent and biologically coherent, suggesting that IL-17 is a key mediator in this process. Together, these results reveal an integrated regulatory mechanism and could identify novel targets for the development of bone anabolic therapies.

## Data Availability

The original contributions presented in the study are included in the article/supplementary material, further inquiries can be directed to the corresponding authors.
